# Bacterial Contamination in Intensive Care Unit at Al-Imam Al-Hussein Hospital in Thi-qar Province in Iraq

**DOI:** 10.5539/gjhs.v5n1p143

**Published:** 2012-11-11

**Authors:** Nazar Edward Nasser, Ali Taher Abbas, Saad L. Hamed

**Affiliations:** 1 College of Science, Al-Mustansiriya University, Baghdad, Iraq; 2 College of Medicine, Thi-qar University, Nasiria, Iraq

**Keywords:** ICU, bacterial contamination, MIC

## Abstract

Cross- infection from patient to patient or from hospital personnel to patients represents constant hazards. It is one of the most important causes of morbidity and mortality especially in Intensive Care Unit all over the world. To identify the types and the source of bacterial contamination in ICU and to study the sensitivity of bacterial isolates to commonly used antibiotics in hospitals this study had been conducted in Al-Imam Al-Hussein hospital in Thi-qar province for the period from the 1^st^ of September to the end of December 2011. A total of 320 swabs and samples were collected from 17 different sites of Intensive Care Unit environment and inoculated on a normal cultural media, then incubated at 37°C for 24 hour. The obtained growth revealed different bacterial colonies which had been tested for their morphological and biochemical characteristics. Sixty eight of pure isolates were obtained including 24 (35.29%) Gram positive bacterial isolates, and 44(64.71%) of Gram negative bacterial isolates, the highest rates (19.11%) of bacterial contamination had been found on the walls and the floor. Sensitivity tests for all isolates were done using 25 types of commonly used antibiotics in Iraq, among Gram negative bacteria and gram positive bacteria the genus *Enterobacter* spp and *Staphylococcus* spp respectively, showed the highest resistance to most of the tested antibiotics, MIC tests for 5 types of antibiotics being applied for the most resistant and the most sensitive isolates had identified that all isolates have a low rate of MIC against Ciprofloxacine. *Bacillus* spp and *Enterobacter* spp were the most prevalent bacterial contaminants of Intensive Care Unit environment. such contamination could be managed mostly by strict application of sterilization measures.

## 1. Introduction

Hospital infection is called nosocomium, occur in about 5% of all admitted patients. In certain health facilities, specifically, Intensive Care Unit (ICU) up to 10% of the patients acquire a Nosocomial infection. An overall of about two million Nosocomial infections occur each year in USA, (Nester et al., 2004, Berriel et al., 2006). The rate of death as a Nosocomial infection in Iran 2003 among hospitalized infants in neonatal Intensive Care Unit of Imam Reza hospital in Mashhad was 17.3% (Naderi, 2007). Khan et al. (2010) found that the rate of Nosocomial urinary tract infection in a teaching hospital in Pakistan was (20.43%). Among gram negative bacteria that cause Nosocomial infection, mostly urinary tract infection, are; *Escherichia coli*, *Klebsiella* spp, *Pseudomonas* spp and *Proteus* spp, while Streptoccoci are among gram positive bacteria that may cause respiratory tract infection, beside *Staphylococcus aureus* and *S. epidermidis* were detected to cause septicemia (Otter & French, 2009). Bacterial contamination in hospitals related directly or indirectly to the improper uses of antibiotics by patients and when the disinfectant are used with concentrations lower than the recommended for cleaning purposes in hospital leading to the appearance of new strains of resistant bacteria to the commonly used antibiotics. Ultimately the patients will need additional treatment and long periods of admission in hospital, which may end with severe side effects (Deep et al., 2004, Dorchis, 2005; Hotchkiss et al., 2007; Vasselle, 2008).

The aim of this study was to identify the types and the source of bacterial contamination in ICU, to study the sensitivity of bacterial isolates to commonly used antibiotics in hospitals and the MIC tests for certain antibiotics.

## 2. Material and Methods

### 2.1 Study Design and Setting

A cross sectional study had been conducted in intensive care unit at Al-Imam Al-Hussein hospital at Thi-qar province, which is one of the southern province in Iraq for the period from 1^st^ of September to the end of December 2011.

### 2.2 Sampling

Three hundred and twenty swabs were collected from the skin of patients, hands of medical staffs, and from different sites related to the devices and utensils used in the ICU including; medical instruments, surgical instruments, sphygmomanometer, sets of intravenous (IV) fluid, masks of O_2_ supplying apparatus, drums, and from the gowns of medical staffs, bed clothes, beside swabs were also taken from the surroundings; floor, walls, windows and door handles, wooden furniture, tables, cabinets, slots of cooling and heating devices, sink, beside samples from the indoor air of the wards of ICU were also taken.

### 2.3 Cultural Media

Swabs were incubated on cultural media; Blood agar, MacConkey agar and Nutrient agar, being prepared according to the manufacturing companies, and incubated at 37°C for (24-48) hours.

### 2.4 Isolation and Identification

Purification of bacterial growth colonies yield pure isolates of bacteria and subsequently their cultural, morphological, microscopical and biochemical characteristics had been studied according to the correlated references (Retty et al., 2007; Alexander & Dennis 2001). For identification of isolates the following kits had been used:

API Staph kit (BioMeriux) for staphylococci identification

API 20E kit (BioMeriux) for Gram -ve bacilli identification

MICEVA kit (Hi media- India) for MIC test

### 2.5 Antimicrobial Sensitivity Tests

Susceptibility for the studied isolates were investigated according to Nester et al. (2004) by using Muller - Hinton agar and the following antibiotics discs:

Cefepime, Piperacillin, Cefotaxime, Gentamicin, Tetracycline, Doxycycline, Ciprofloxacine, Ofloxacin, Levofloxacin, Nalidixic acid, Oxacillin, Vancomycin, Erythromycin, Rifampin, Clindamycin, Ampicillin, Cephalothin, Ceftazidime, Imipenem, Aztreonam, Amikacin, Chloramphenicol, Ceftriaxon, Ticarcillin - Clavulanic acid and Amoxicillin - Clavulanic acid.

The test of MIC had been measured by using Ceftriaxon and Meropenem powder utilized two fold dilution method on Muller - Hinton agar, and Ciprofloxacin, Piperacillin-tazobactum and Amikacin with MICEVA kit, then results were recorded according to CLSI (2007).

## 3. Results and Discussion

Bacterial growth had been observed in 57 cultures (17.8%) out of 320 swabs and samples which was collected from 17 sites distributed in ICU environment (Table 1).

**Table 1:** The positive bacterial growth cultures and the pure isolates in ICU environment

Sites (20 swabs for each site)	Positive Growth Cultures		Pure isolates	

	No.	%	No.	%
Doors & windows	0	0	0	0
Bed	3	5.3	4	20
Table	2	3.5	2	10
Cabinate	6	10.6	7	35
Walls & Floor	9	15.8	13	65
Slots of cooling and Heating device	1	1.7	1	5
Wood furniture	0	0	0	0
Sink	8	14	9	45
Medical apparatus	8	14	10	50
Masks of O_2_ supplying apparatus	2	3.5	3	15
Set of intravenous (IV) fluid	1	1.7	1	5
Sphygmomanometer	2	3.5	2	10
Gowns	1	1.7	1	5
Hands of medical staff (10 swabs)	1	1.7	1	5
Surgical instrument	3	5.3	3	15
Patient skin	6	10.6	6	30
Ward air (10 swabs)	4	7	5	25
	57	100	68	

The most evident contamination sites found in the ICU environment were the walls and floor revealed in 13 isolates (19.11%) followed by medical apparatus, 10 isolates (14.7%) of the total isolates, yet the lowest level of contamination was 1 isolate (1.47); at the set of IV fluid, hands of medical staff and their gowns, and slots of cooling and heating devices, while no contamination was observed on doors and windows and wooden furniture. Table 2 shows the distribution of the pure cultures according to their sites and type of genus.

**Table 2:** Distribution of pure isolates on the sites and types of Bacteria

Genus	1*	2	3	4	5	6	7	8	9	10	11	12	13	14	15	16	17
*Staphylococcus aureus*														+	+		+
*Staphylococcus chromogenes*													+			+	
*Staphylococcus epidermidis*																+	+
*Staphylococcus haemolyticus*																+	
*Bacillus subtilis*						+											
*Bacillus cereus*			+	+	+	+		+	+	+							
*Enterobacter cloacae*		+	+		+				+		+	+					
*Enterobacter sakazaki*									+								
*Bordetella spp.*				+	+	+			+								
*Pantoea spp.*		+															
*Klebsiella pneumonia*			+		+					+							
*Citrobacter freundi*						+			+								
*Citrobacter yongae*					+												
*Escheria hernanni*					+												
*Escherichia coli*						+											
*Pseudomonas Aeruginosa*												+					
*Proteus mirabilis*										+							
*Rahnella aguatilis*			+														

1* .Doors & windows 2. Bed clothes 3. Table 4. Cabinet 5. Walls & Floor 6. Sink 7- Wooden furniture 8- Slots of cooling & heating device 9-Medical apparatus 10-Mask of O_2_ supply 11-Set of IV fluid 12- Sphygmomanometer 13- Gowns 14-Skin of palm of medical staff (only 10 samples) 15- Surgical instrument 16- Skin of patient 17- Ward air

The pure cultures were divided into two groups depending on Gram stain, accordingly 24 Gram positive isolates and 44 Gram negative isolates were identified (Table 3).

**Table 3:** Numbers and percentage of pure isolates in the studied samples

Bacteria	No.	%	Type	
*Staphylococcus spp.*	6	**25**	**Gram positive**
*Bacillus spp.*	18	**75**	**Gram positive**
*Enterobacter spp.*	15	34	Gram negative
*Bordetella spp.*	8	18.2	Gram negative
*Pantoea spp.*	6	13.6	Gram negative
*Klebsiella pneumonia*	4	9.1	Gram negative
*Citrobacter spp.*	4	9.1	Gram negative
*Escheria hernanni*	1	2.3	Gram negative
*Escherichia coli*	3	6.8	Gram negative
*Pseudomonas aeruginosa*	1	2.3	Gram negative
*Proteus mirabilis*	1	2.3	Gram negative
*Rahnella aguatilis*	1	2.3	Gram negative
	68		

The most prevalent genus among Gram positive bacteria was *Bacillus* spp. (18 isolates) found in 7 out of 17 sites, while the most prevalent genus among Gram negative was *Enterobacter cloacae* (15 isolates). On the other hand 6 isolates of *Staphylococcus* spp. (25%) among Gram positive bacteria were identified, being also found by (Manges et al., 2001), while *E. coli* represent only 6.8% of total Gram negative bacteria which was inconsistence with a study done in Erbil (Muhammed, 2002) where an extremely high percentage (46.21%) of contamination with this species was found, this may be due to the differences of the sites of swabs being taken from the environment of the hospital as a whole in Erbil or may be explained by the level of health awareness of both, patients and health staff in different communities (Delzell & Lefevre, 2000). On the other hand *Pseudomonas aeruginosa* represent only 2.3% of total gram negative bacteria, which is an important Nosocomial pathogen invasive, toxigenic, multi-drug resistant (Greenwood et al., 2007) and found to be responsible about 28.5% of ICUs Nosocomial infection in Mombia, India (Pal Ramprasad, 2010).

Susceptibility tests for some antibiotics showed different results depending on the genus of bacteria and type of antibiotic. For *Enterobacter* spp. the resistance was statistically highly significant against 7 antibiotics, p value<0.01 (Ampicillin, amoxicillin clavulanic acid, Cephalothin, Imipenem, Ciprofloxacin, Levofloxacin and Ofloxacin) while it was significant for 5 antibiotics with p value <0.05 (Piperacillin, Ticarcillin clavulanic acid, Cefepime, Ceftriaxon and Aztreonam), yet it was insignificant, p value >0.05 against 7 antibiotics (Cefotaxim, Ceftazidime, Gentamycin, Amikacin, Tetracycline, Nalidixic acid and Chloramphenicol).

Among Gram positive bacteria, susceptibility tests conducted for *Staphylococcus* spp. showed resistance which was statistically highly significant against 6 antibiotics with p value < 0.01 (Ampicillin, Cefepime, Ceftazidime, Imipenem, Chloramphenicol and Oxacillin), while it was insignificant, p value >0.05 against 15 antibiotics (amoxicillin clavulanic acid, Tetracycline clavulanic acid, Cephalothin, Cefotaxime, Ceftriaxon, Gentamycine, Amikacin, Tetracycline, Ciprofloxacine, Levofloxacin, Ofloxacin, Clindamycin, Rifampin, Erythromycin and Vancomycin).

The appearance of resistance for β-lactamase antibiotics specifically amoxicillin and to a lower extent Piperacillin could be related to many causes; production of β lactamase enzymes and its effect which lead to the breakdown of the β – lactame cycle in penicillins and cephalosporines changing it into inactive compounds (Pfeillf et al., 2000), or may be because of the changes being occurred in the porins of the cellular membrane and ultimately its effect on the cell permeability (Nester et al., 2004), some Gram negative bacteria are resistant for β–lactame antibiotic because it have an Efflux pump system which lead to pump the antibiotics from intracellular to extracellular space (Schweizer, 2003).

The gradual increase in the resistant of Enterobacteriaceae against β-lactame antibiotics (1^st^ and 2^nd^ generation of pencillins and cephalosporines) reduce the efficacy of these antibiotics in eradicating diseases of bacterial etiology completely since these resistance will lead to continuous change in the epidemiology of these disease (Sahm et al., 2001), while the effect of Extended Spectrum β-lactamase (ESBLs) became more evident against the 3^rd^ generation of penicillins and cephalosporines (Woodford et al., 2006)

The resistant against recently introduced β-lactame antibiotic; Aztreonam is related to many causes; it`s sensitivity for β-lactamases enzyme produced by *Proteus mirabilis*, *Klebsiella* *pneumoniae*, and *E.coli*, or may be due to the weak affinity of antibiotic to the penicillin binding proteins in cell wall (Brown & Amyes, 2006).

The high sensitivity of the studied isolates for Imipenem belong to Carbapenems group, one of the recently used antibiotic, could be due to its limited use in Iraq. Although resistant was also recorded among 4.41% of these isolates, and the cause could be related to the development in the mechanism of bacterial resistance such as its production for Carbapenemases enzymes related to β - lactamases enzymes type B and D (Laclero et al., 1999).

One of three mechanisms may explain the resistance of some bacteria against aminoglycosides antibiotics; production of converted enzymes which inhibit the activity of antibiotics, changing the target of antibiotics, or through the change of the permeability for the cell barrier (Levinson & Jawetz, 2004, Nester et al., 2004).

The test of MIC was applied to detect the lowest concentration of a specific drug that prevents the growth of an organism in vitro. Interpreting the significance of a given MIC requires knowledge of the level of the drug that can be reached in the patient. On the other hand MIC can also used to determine whether the resistant of a microorganism is increased against specific antibiotic and if it is sufficiently susceptible or not to it to achieve successful treatment (Nester et al., 2004).

The results of MIC tests showed that the lowest concentration of Ciprofloxacin was 0.016 µm/ml (Table 4), to exert an effect on *Enterobacter* spp., *E. coli*, *Citrobacter* spp. and *Pantoea* spp. The lowest concentration of Piperacillin tazobactum was 0.25 µm/ml against *Enterobacter* spp., and Amikacin against *Bacillus* spp., while the lowest concentration of Ceftriaxone was 1 µm/ml against *Bordetella* spp., for imipenem was 0.05µm/ml against *Bordetella* spp., *Pantoea* spp., *Staphylococcus* spp., and *Bacillus* spp.

**Table 4:** MIC test of five antibiotics with the studied isolates

Bacteria	Ciprofloxacine	Piperacillin-tazobactum	Amikacin	Ceftriaxon	Meropenem
*Enterobacter spp.*	3 - 0.016	96 – 0.25	64 – 0.5	1024 – 2	16 – 0.5
*Klebsiella pneumonia*	3 – 0,125	48 – 16	4 – 0.75	1024 – 8	1
*Escherichia coli*	3 - 0.016	48 – 16	256 – 1.5	256 – 4	2 – 1
*Citrobacter spp.*	2 - 0.016	256 – 4	64 – 0.38	1024 – 8	2 – 1
*Bordetella spp.*	0.25-0.023	192 – 16	16 – 0.75	1024 – 1	4 – 0.5
*Pantoea spp.*	0.094-0.016	321 – 12	1.5 - 0.5	1024 – 4	4 – 0.5
*Proteus mirabilis*	1	1.5	32	2	16
*Pseudomonas aeruginosa*	0.19	12	3	1024	32
*Escheria hernanni*	0.125	128	96	32	2
*Rahnella aguatilis*	1	8	2	512	4
*Staphylococcus sp.*	1 – 0.25	48 – 8	4 – 0.75	1024 – 4	4 – 0.5
*Bacillus spp.*	1 – 0.094	256 - 2	4 - 0.016	1024 - 16	4 – 0.5

## 4. Conclusions

*Bacillus* spp and *Enterobacter* spp were the most prevalent bacterial contaminants of ICUs environment predominantly at the walls and the floor, on the other hand *Enterobacter* spp show a high resistance to commonly used antibiotics. Gradual increase in the resistant of microbes to previously and recently produced antibiotics may interfere with the tremendous effort provided by health facilities to control the spread of microbial disease in the community. This problem could be controlled to some extent by restriction of purposeless uses of antibiotics and by eliminating contamination in the environment of hospitals by applying strict quality standards concerned with the hygienic manners both of patients and health staff, and the performance of invasive procedures using aseptic technique.
